# Context-dependent consumer control in New England tidal wetlands

**DOI:** 10.1371/journal.pone.0197170

**Published:** 2018-05-17

**Authors:** Alexandria Moore

**Affiliations:** School of Forestry and Environmental Studies, Yale University, New Haven, CT, United States of America; Maurice Lamontagne Institute, CANADA

## Abstract

Recent studies in coastal wetlands have indicated that consumers may play an important role in regulating large-scale ecosystem processes. Predator removal experiments have shown significant differences in above-ground biomass production in the presence of higher level consumers, or predators. These results indicate that predators play an important role in regulating biomass production, but the extent to which this regulation impacts additional ecosystem functions, such as nutrient cycling and organic matter accumulation, is unclear. This study evaluated the impact that consumers have on large-scale ecosystem processes within southern New England tidal wetlands and contributes to the general understanding of trophic control in these systems. I established enclosure cages within three coastal wetlands and manipulated the presence of green crab predators to assess how trophic interactions affect ecosystem functions. Findings suggest that although these consumers may exert some top-down effects, other environmental factors, such as other consumers not studied here or bottom-up interactions, may variably play a larger role in the maintenance of ecosystem processes within the region. These results indicate that the loss of top-down control as an important mechanism influencing ecosystem functions may not hold for all wetlands along the full extent of the New England coastline.

## Introduction

Research in a wide variety of ecosystems has shown that trophic structure and species interactions are important determinants of ecosystem function [1–10]. The loss of large apex predators within aquatic ecosystems, for instance, may impact phytoplankton density, affecting primary production, CO_2_ uptake rates, and the direction of carbon flux between lakes and the atmosphere [11–12]. These and other studies show that trophic interactions may be important mediators of large-scale ecosystem processes, but the strength and degree to which these interactions influence ecosystem functions is often dependent upon several variables, including habitat type, community structure, abiotic factors, and anthropogenic impacts [13–15].

In wetland ecosystems, the reigning ecological paradigm has historically focused on the importance of bottom-up control (predominance of nutrient supply) over top-down control (predominance of top predator effects) in regulating key ecosystem functions and services [16–19]. Yet, recent research in tidal salt marshes suggests that top-down factors, particularly the presence or absence of predator species, may have significant impacts on ecosystem functions within these systems [20–26].

Along the northern New England coastline, the loss of native marine predators like blue crabs (*Callinectes sapidus*) and striped bass (*Morone saxatilis*) due to intense recreational fishing has released the herbivorous purple marsh crab (*Sesarma reticulatum*) from consumer control [21]. This, in turn, has precipitated large-scale reductions in salt marsh area via overgrazing and soil destabilization, indicating that top-down consumer-driven processes play a much larger role in regulating the stability of these ecosystems than was previously thought [23–26]. Such research challenges the historical notion that bottom-up factors exclusively regulate large-scale processes within wetland ecosystems. However, the generality of such results has been limited in regional extent (i.e. northern vs southern New England) and in the evaluation of ecosystem functions beyond primary production. Therefore, the purpose of this study was to contribute to this general understanding of top-down versus bottom-up control within salt marshes by evaluating the impact that consumers have on several large-scale ecosystem processes. Here, I report on the experimental evaluation of the role that consumer species play in determining above-ground biomass production and soil quality within several southern New England tidal salt marsh ecosystems. Findings show that although the consumers studied here may exert some top-down effects, other environmental factors may variably play a larger role in the maintenance of ecosystem processes. These results indicate that the recent consensus on top-down control as the key driver maintaining ecosystem processes may not hold in all wetlands along the full extent of the New England coastline.

## Materials and methods

### Study system and hypotheses

This study was conducted at three sites along the Connecticut shoreline of Long Island Sound on the northeast coast of the United States. Tidal salt marsh communities here are dominated by salt-tolerant grasses, including smooth cordgrass (*Spartina alterniflora*), and myriad detritivore, herbivore, and predator species. Marsh landscapes are most directly impacted by a handful of crustacean consumers, including fiddler crabs (*Uca pugnax*) and purple marsh crabs (*Sesarma reticulatum*). The fiddler crab is a detritivore that alters the landscape through its burrowing behavior and deposit feeding (e.g. sieving through sediment particulates for organic matter) [27, 28]. The purple marsh crab is a burrowing herbivore that directly consumes marsh vegetation above- and below-ground [22]. Together, the natural behaviors of these two species may contribute to ecosystem functions, such as biomass production and soil quality, within salt marsh ecosystems (Table 1). In particular, fiddler crabs acquire food by continuously sifting through sediment for algae, microbes, fungus, and decaying detritus. This behavior may decrease soil organic matter content, increase soil inorganic nitrogen availability, and increase the rate of change in soil inorganic nitrogen (a proxy for mineralization rate) as the top layers of soil are constantly turned-over. Additionally, the maintenance of crab burrows improves soil drainage, oxygenating marsh sediments, thus increasing decomposition of plant-generated below-ground debris [28]. Primary production may then be positively impacted by fiddler crab detritivory as a result of improved soil conditions [28–31]. Purple marsh crabs, on the other hand, can directly decrease plant productivity through herbivory, while also improving soil conditions through burrowing behavior [24, 29–32]. The net reduction in plant biomass due to herbivory may also mean that less plant material is left to enter the detrital chain and overall soil organic matter content is reduced.

**Table 1:** Predicted impact of fiddler crabs and purple marsh crabs on measured variables.

	Burrow density	Soil organic matter	Soil nitrogen content	Rate of change in soil nitrogen	Above-ground biomass
Fiddler crabs	+	-	+	+	+
Purple marsh crabs	+	-	+	+	-
Combined effect	+	-	+	+	+/-

The positive symbol indicates a predicted increase in the measured variable relative to control conditions where these species are absent while the negative symbol indicates a predicted decrease in the measured variable relative to the same control conditions.

Fiddler crabs and purple marsh crabs are also important dietary prey of the European green crab (*Carcinas maenas*, hereafter ‘green crab’), a non-native and non-burrowing species found along the New England coast. Introduced in the 1800s, the green crab has become an important predator within these coastal marshes, supplanting native predators that have experienced reduced population sizes [21, 33]. The presence or absence of this predator may moderate the impact that its prey has on salt marsh ecosystem processes. In particular, if the presence of green crab predators leads to reductions in the functional density of fiddler crabs and purple marsh crabs, then the key processes that these prey species affect should be similarly altered (Table 2). Specifically, green crab predators should facilitate an increase in soil organic matter content, a decrease in soil inorganic nitrogen content and the rate of change in soil nutrient content, and an increase above-ground biomass production (under the assumption that herbivory more strongly influences vegetation growth compared to improved soil conditions).

**Table 2:** Predicted impact of predator addition (“Pred”) and predator exclusion (“No pred”) treatments on measured variables.

	Burrow density	Soil organic matter	Soil nitrogen content	Rate of change in soil nitrogen	Above-ground biomass
Pred	-	+	-	-	+
No pred	+	-	+	+	-

The positive symbol indicates a predicted increase in the measured variable relative to control conditions while the negative symbol indicates a predicted decrease in the measured variable relative to control conditions.

### Study sites

I conducted field experiments between May and August 2015 in three tidal salt marshes situated along 20 miles of the Connecticut coastline: Farm River State Park in East Haven, CT, USA (41°15’21.82”N, 72°51’24.12”W), Fence Creek marsh in Madison, CT, USA (41°16’33.25”N, 72°35’10.24”W) and Hammonasset Beach State Park in Madison, CT, USA (41°15’59.88”N, 72°33’30.30”W). This time period covers the growing season when each target species was most active. These sites exhibited evidence of reduced predator populations as determined by observation of the extent of cordgrass overgrazing and the recession of the low marsh. Fiddler and purple marsh crab population densities were high along the full extent of the marsh as determined by observations and pitfall traps placed adjacent to each of the designated experimental plot areas (S1 Table). Pitfall traps were constructed using 2.5-quart plastic buckets and 7.5 cm diameter open-top plastic cylinders (empty tennis ball cans) with drainage holes drilled in the bottom and sunk until the top edge was flush with the marsh substrate. Eight traps, four of each type, per site were placed >2m apart and left unbaited for 24 hours in May 2015 and checked the following day at low tide. Predator crabs were targeted using one baited and one unbaited Quonset crab pots placed adjacent to experimental treatment plots along the salt marsh creek edge where the tide was guaranteed to submerge the traps during the tidal regime. Pots were deployed overnight in each site on five separate occasions in May 2015 and were largely unsuccessful in collecting predator species, indicative of reduced populations within the region (S1 Table).

### Ethics statement and permits

Research in each of the study sites was approved by the Connecticut Department of Energy and Environmental Protection (DEEP). The Certificate of Permission (certificate number: 201503241-KR) specifies access and use of Farm River State Park, Fence Creek Marsh, and Hammonasset Beach State Park for the research and non-vertebrate animal use described below. As coastal tidal wetlands, each site included in this study is defined as protected and use was limited to those specified in the following experimental methods. No protected species were sampled for this study.

### Enclosure experiment

Within each wetland, I established experimental plots in groups of four (two manipulation plots, two non-manipulation control plots) and randomly placed these groups along the creek-bank edge of the low marsh where smooth cordgrass was the dominant plant. Each site contained 24 plots representing 6 replicates of the four treatments with the exception of Hammonasset Beach State Park, where treatments could only be replicated 5 times due to space limitations. Manipulation plots were constructed using 0.6cm x 0.6cm galvanized wire cloth formed into a circular cage standing approximately 1m tall with a 1m^2^ basal area. One non-manipulation control plot in each grouping was identical to manipulation plots except along opposing sides of each cage, a 0.5m x 0.5m opening was made to allow for access to all target species. The purpose of this plot was to evaluate the effect that the cage itself might have on the outcome of the experiment. All cages were then sunk into the marsh surface to a depth of approximately 0.3m, leaving 0.5x 0.2m openings exposed in the non-manipulation cage control. The remaining non-manipulation control was an open-field plot with no cage and was used to evaluate baseline site conditions to which manipulation plots were compared. This overall arrangement ensured that all non-predatory target species (i.e. fiddler crabs and purple marsh crabs) could move freely through cages by either crawling between/above the wire mesh or via burrowing underneath the sunken cage, while target predator species were either fully limited from or fully confined to manipulation cages.

Manipulation plots were then assigned to one of two treatments: (1) Predator Exclusion and (2) Predator Addition. The Predator Exclusion plots were unaltered and prevented access to cages by the target predator species. The Predator Addition plots were stocked at average field density (three individuals per cage) as determined by the literature for the region [34, 35] using green crab individuals purchased from local bait and tackle shops during blackfin tuna fishing season in June 2015. Green crabs used for stocking were all adults with a carapace width ranging from 50mm to 75mm and were not individually sexed. Within all plots, three holes created from initial soil samples were augmented to a diameter of 10cm and a depth of 20cm using a hand trowel. In the Predator Addition plots, these augmented holes served as burrows and desiccation refuge for the non-burrowing green crab individuals.

### Measurements and laboratory analyses

All measurements, with the exception of above-ground biomass and the rate of change in soil nitrogen, were taken prior to the onset of the experiment in May to determine initial conditions and again at the end of the experimental period in August to evaluate treatment effects. Biomass and the rate of change in soil nitrogen were only evaluated at the end of the experimental period in May.

Burrow density was used as a proxy for population density for fiddler crabs and purple marsh crabs and is the standard measure used given the difficulty of accurately evaluating number of individuals within a given area [36, 37]. I determined the burrow density by counting the number of burrow holes within the confines of each 1m^2^ experimental plot, without differentiating between burrows made by fiddler and purple marsh crabs.

I determined soil organic matter content (SOM) using the standard loss-on-ignition (LOI) method [38]. Three soil samples were collected from each plot using a hand trowel to form a 5cm diameter core to a depth of 20cm. Soil samples from each plot were homogenized and oven-dried at 105°C for 24 hours or to constant weight. Dried soil samples were then weighed in porcelain crucibles (pre-weighed) and placed in a muffle furnace at 500°C for at least 16 hours. Crucibles were cooled in a desiccator over calcium chloride and re-weighed. The LOI content of samples were calculated as:

$$LOI,\%=\frac{{Weight}_{105}-{Weight}_{500}}{{Weight}_{105}}\times 100$$

I measured soil inorganic nitrogen concentration using a potassium chloride (KCl) extraction method [39]. Soil samples were collected from each plot using a hand trowel to form a 5cm diameter core. In the field, a 10g subsample of these cores was immediately placed into prepared centrifuge tubes containing 25 mL of 2M KCl and shaken vigorously until well mixed. Extracting soluble nitrogen in the field halted microbial processing of the nitrogen. Bottles were stored in a cooler until transported to Yale laboratory facilities where they were refrigerated overnight to allow the solution to separate and analyzed further the following day. After separation, the supernatant was decanted and frozen until analysis. Solutions were then thawed and analyzed for total extractable nitrogen content using a flow analyzer (Astoria 2: Astoria-Pacific, Inc).

The rate of change in soil nitrogen was determined at the end of the experimental period in August using a 31-day incubation followed by a KCl extraction [39, 40]. Use of this method allows for a more accurate evaluation of treatment effects by minimizing the impact of potentially confounding *in situ* variation in abiotic conditions, such as temperature and soil moisture [40]. Soil samples were collected from each plot using the aforementioned method. Total nitrogen content of soil representing Day 0 of the analysis was measured using the KCl method described above upon sampling. An additional 20g of soil from each plot sample was added to pre-weighed and labeled plastic urine cups. This additional sample was used to determine the total nitrogen content of the soil at the end of the 31-day period. Each cup was covered loosely in plastic wrap and sealed with a rubber band, allowing for air diffusion but maintaining moisture content. Urine cups were placed in an incubator at 20°C and maintained at 65% moisture content, as determined by testing for the water holding capacity of each sample, for 31 days. On Day 31, a 10g subsample of each incubated soil sample was taken and total nitrogen content was determined using the KCl method. The rate of change in soil nitrogen for each plot was determined by calculating the difference between nitrogen content at Day 0 and Day 31 and dividing by the number of days [40].

Above-ground biomass was evaluated at the end of the growing season in August. All standing vegetation within each plot was collected by cutting plants at the ground level using garden shears. All non-plant material was removed from vegetation prior to being placed in labeled paper bags and immediately transported to Yale laboratory facilities. Bags were then left out to air dry for several weeks before obtaining a dried weight using a top-loading scale [41].

### Effect sizes and statistical analyses

The effect size for each response variable was calculated to quantify the magnitude and direction of manipulation treatment impacts. Here, effect size was defined as ln(*X*_*e*_*/X*_*c*_) where *X*_*e*_ and *X*_*c*_ are the mean manipulation treatments and non-manipulation control ecosystem function response, respectively [42–44]. An effect size ln(*X*_*e*_*/X*_*c*_) > 0 means that treatments had a positive impact on the measured ecosystem function relative to the control, ln(*X*_*e*_*/X*_*c*_) ~ 0 means that manipulations had no significant impact, and ln(*X*_*e*_*/X*_*c*_) < 0 means that treatments had a negative impact on the measured ecosystem function relative to the control. These values were not included in statistical analyses and were instead produced to highlight treatment effects to investigate further.

I analyzed burrow density, organic matter, soil nitrogen, and above-ground biomass using linear models and the rate of change in soil nitrogen was evaluated using a generalized linear mixed-effects model (GLMM). For each model treatment and site were set as fixed effects with initial conditions as a covariate, and block (i.e. treatment groupings) was set as the random effect nested within site. This nesting allowed me to address any potential autocorrelation arising from nonindependence among treatment groups. Each model also included a treatment by site interaction term to identify site-specific differences. For the model constructed for the rate of change in soil nitrogen, the error distribution was visually estimated using histograms and Q-Q plots fit to several known potential distributions and the goodness of fit for those with the closest visual match was evaluated using the “fitdistrplus” library and Kolmogorov-Smirnov test. The GLMM for the rate of change in soil nitrogen was then fit to a gamma error distribution using a log link. For response variables that produced significant interaction effects between treatment and site, additional models for individual sites were constructed to determine context-dependency of treatment effects. For these, the aforementioned fixed and random effects were used but “site” was removed from the model. Akaike information criterion (AIC) scores and the fit of the data were used to select the best model for each response variable. I analyzed the models using the “lme4” library [45] along with the “lmerTest” [46] and “multcomp” libraries [47] to get significance estimates. When significant relationships occurred, I performed Tukey contrasts to determine which means were significantly different. Each response variable was also analyzed using a one-way ANOVA to test for differences between the non-manipulation open plot and the non-manipulation cage plot. All statistical analyses were conducted in RStudio (v. 1.0.136).

## Results

### Non-manipulation controls

For all response variables between and among sites, the cage control showed no difference from the open control (S2 Appendix). These two categories were therefore combined to create one main control treatment with which to evaluate differences between manipulation treatments.

### All sites combined

#### Burrow density

**Fig 1:**
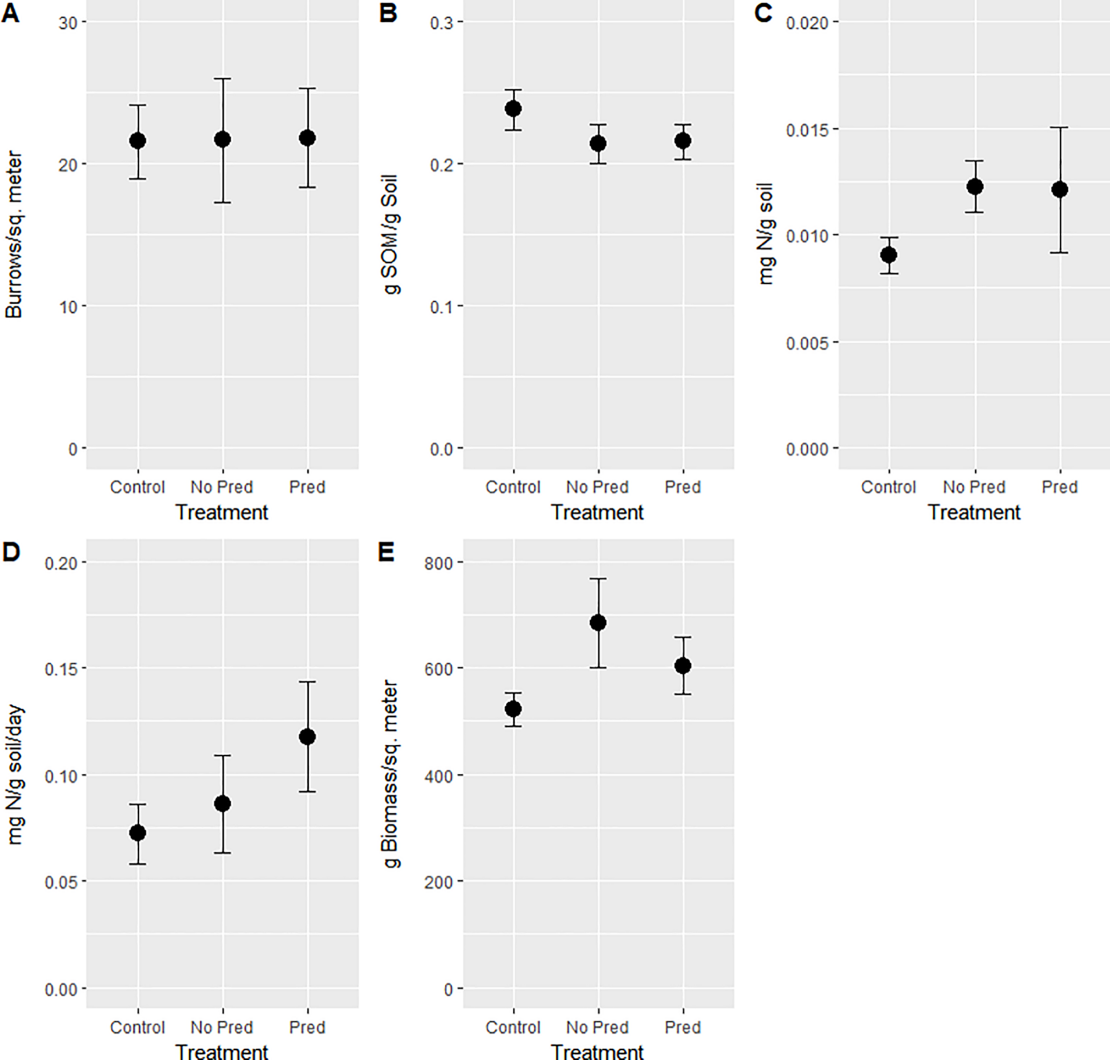
Response variable by treatment at the end of the experimental period. (A). Burrow density. (B) Soil organic matter (SOM). (C) Soil inorganic nitrogen content. (D) Rate of change in soil inorganic nitrogen. (E) Above-ground biomass. Error bars indicate one standard error.

The Predator Exclusion treatment (hereafter ‘No Pred’) and Predator Addition treatment (hereafter ‘Pred’) were not significant predictors of burrow density relative to the control (p>0.4, Table 3, Fig 1A). When compared to one another, the manipulation treatments also did not differ with respect to burrow density (p>0.9).

**Table 3:** Effect size for each response variable with all site combined.

	Burrow density	Soil organic matter	Soil nitrogen content	Rate of change in soil nitrogen	Above-ground biomass
Pred	0.01245	-0.10163	0.28768	0.48550	0.14719
No Pred	0.00416	-0.10629	0.30421	0.17768	0.27139

Effect size was defined as ln(*X*_*e*_*/X*_*c*_) where *X*_*e*_ and *X*_*c*_ are the mean manipulation treatments and non-manipulation control ecosystem function response, respectively. Underlined values indicate marginal differences (p < 0.1) from the control.

#### Soil organic matter

The No Pred and Pred treatments were not significant predictors of SOM relative to the control (p>0.1, Table 3, Fig 1B). Manipulation treatments also did not differ in terms of soil organic matter (p>0.9).

#### Soil inorganic nitrogen

The No Pred and Pred treatments were not significant predictors of the change in soil nitrogen relative to the control (p > 0.5 Table 3, Fig 1C). The No Pred and Pred treatments did not differ statistically (p>0.4).

#### Rate of change in soil inorganic nitrogen

The No Pred and Pred treatments were not significant predictors of the change in soil nitrogen relative to the control (p>0.1, Table 3, Fig 1D). Manipulation treatments also did not differ in terms of the rate of change in nitrogen content (p>0.2).

#### Above-ground biomass

The No Pred treatment had marginally more above-ground biomass (p = 0.0553) while the Pred treatment exhibited no difference compared to the control (p>0.4). The No Pred and Pred treatments did not differ (p>0.6, Table 3, Fig 1E).

### Site by site

#### Farm River State Park

**Fig 2:**
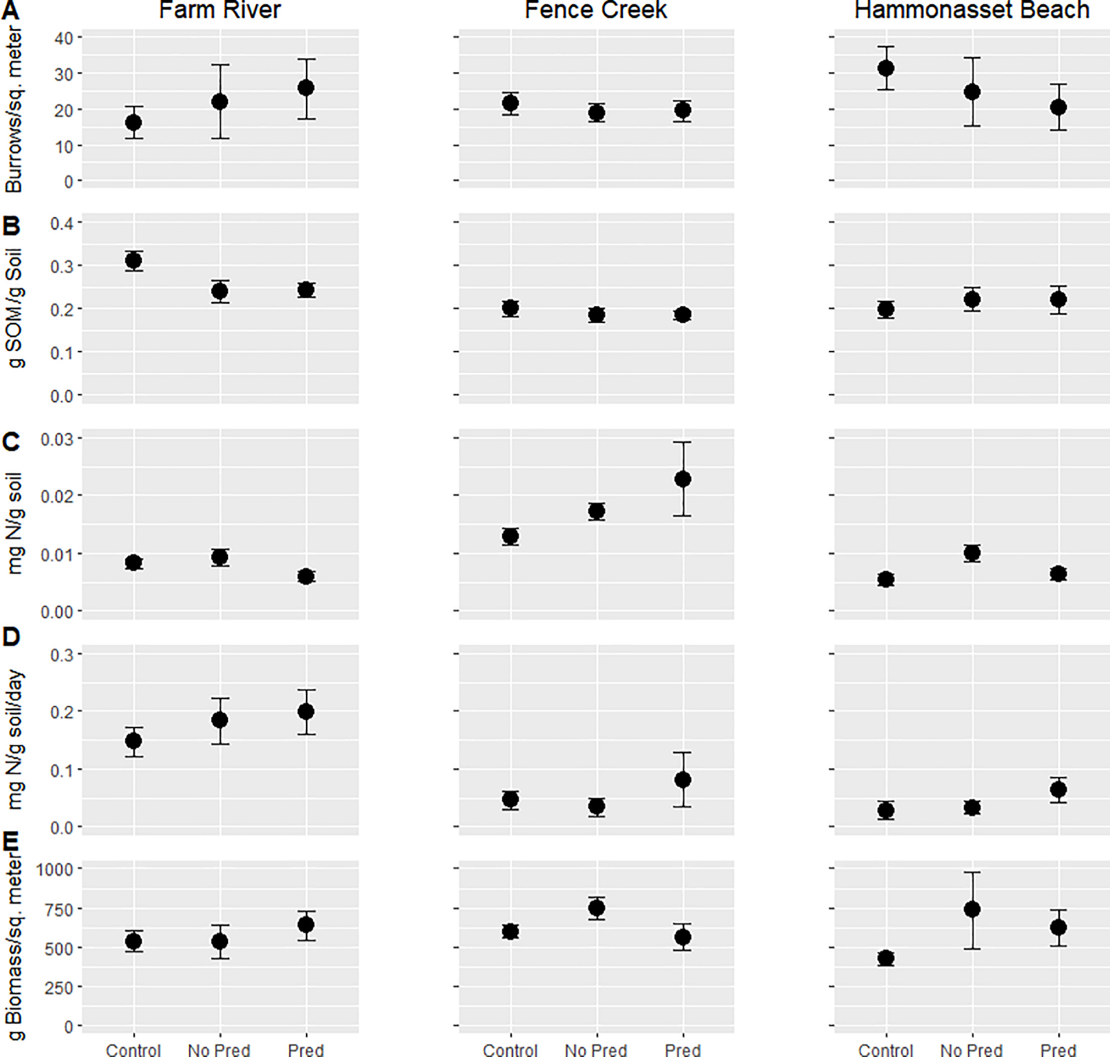
**Response variable by treatment at the end of the experimental period at Farm River State Park, Fence Creek, and Hammonasset Beach State Park** (A). Burrow density. (B) Soil organic matter (SOM). (C) Soil inorganic nitrogen content. (D) Rate of change in soil inorganic nitrogen. (E) Aboveground biomass. Error bars indicate one standard error.

Burrow density was not affected by the No Pred treatment compared to the control but was marginally higher under the Pred treatment (p>0.9 and p = 0.0546, respectively, Table 4, Fig 2). Manipulation treatments did not differ with respect to any of the response variables at this site (p>0.1). SOM was significantly lower in both the No Pred and Pred treatments compared to the control (p = 0.0003 and p = 0.0021 respectively, Table 4, Fig 2). Soil nitrogen was marginally lower in the Pred treatment (p = 0.0803) compared to the control and was significantly lower compared to the No Pred treatment (p = 0.0412, Table 4, Fig 2). There was no difference in soil nitrogen between the No Pred and control treatments. There was no treatment effect on either the rate of change in soil nitrogen or above-ground biomass at this site (p>0.3, Table 4).

**Table 4:** Effect size for each response variable at each individual site.

	Burrow density		Soil organic matter		Soil nitrogen content		Rate of change in soil nitrogen		Above-ground biomass	
	Pred	No Pred	Pred	No Pred	Pred	No Pred	Pred	No Pred	Pred	No Pred
Farm	0.46090	0.31326	**-0.24089**	**-0.25900**	-0.50074	**-0.06867**	0.29650	0.21514	0.17459	-0.0053
Fence	-0.10234	-0.12854	-0.07883	-0.08355	**0.59079**	0.33576	0.57858	-0.30316	-0.06123	0.22007
Hamm	-0.42305	-0.23584	**0.10070**	0.10768	0.38865	**0.50602**	0.82685	0.151308	0.38158	**0.54598**

Effect size was defined as ln(*X*_*e*_*/X*_*c*_) where *X*_*e*_ and *X*_*c*_ are the mean manipulation treatments and non-manipulation control ecosystem function response, respectively. Underlined values indicate marginal differences (p < 0.1) and bold values indicate significant differences (p < 0.05) from the control.

#### Fence Creek

Treatment effects were only observed on soil inorganic nitrogen content and above-ground biomass. The No Pred treatment had marginally more soil nitrogen compared to the control (p = 0.05872, Table 4, Fig 2). The Pred treatment had significantly greater soil nitrogen compared to the control (p = 0.00568), but did not differ compared to the No Pred treatment (p>0.6, Table 4, Fig 2). Neither the No Pred nor the Pred treatments differed from the control (p>0.1) in terms of biomass, but biomass was marginally higher in the No Pred treatment compared to the Pred treatment (p = 0.091, Table 4, Fig 2).

#### Hammonasset Beach State Park

Burrow density was marginally lower in the Pred treatment compared to both the control and No Pred treatments (p = 0.0573 and p = 0.0667 respectively, Table 4, Fig 2); SOM in the No Pred treatment and the control did not differ significantly (p>0.9). SOM in the No Pred and Pred treatments was marginally (p = 0.071) and significantly (p = 0.034) greater than that in the controls, respectively (Table 5, Fig 2). The manipulation treatments did not differ with respect to SOM (p>0.9). Soil inorganic nitrogen was significantly higher in the No Pred treatment compared to both the control (p = 0.0057) and the Pred treatment (p = 0.015, Table 4, Fig 2) but the Pred treatment did not differ from the control (p>0.9). There was no treatment effect on the rate of change in soil nitrogen. Finally, the No Pred treatment had significantly more above-ground biomass than the control (p = 0.00224, Table 4, Fig 2) but did not differ when compared to the Pred treatment (p>0.3), which did not differ from the control treatment (p>0.1).

**Table 5:** Ecosystem variables: Predictions and observations by experimental treatment.

Ecosystem property	Soil organic matter		Soil nitrogen		Rate of change in soil nitrogen		Above-ground biomass	
	Pred	No Pred	Pred	No Pred	Pred	No Pred	Pred	No Pred
Prediction	+	-	-	+	-	+	+	-
Observation	-/0/+	-/0/+	-/+/0	+	0	0	0	0/0/+

The positive symbol indicates an increase in the response variable relative to control conditions while the negative symbol indicates a decrease in the response variable relative to control conditions. Zero indicates no change relative to the control treatment. Cells with mixed symbols represent observed variation in response variable at each site in the following order: Farm River, Fence Creek, and Hammonasset Beach State Park. Cells of observed responses with one symbol indicates that each site responded the same way.

## Discussion

In coastal wetland ecosystems, the reigning ecological paradigm has historically focused on the importance of bottom-up rather than top-down control in regulating key ecosystem functions and services [16–19]. However, recent studies have shown that consumer control plays a significant role in the regulation of functions within these dynamic coastal ecosystems [20–26]. The results of this study contribute additional insight to this growing consensus by showing that the role of consumers, while not insignificant, may vary in importance depending on environmental context.

### Predator impacts on ecosystem functions

#### All sites

Burrow density is a commonly-used proxy for population density for fiddler and purple marsh crabs due to the difficulty of assessing the per capita population size of these species [36, 37]. Burrow density did not differ between any of the treatments at the end of the experimental period when combining all sites (Fig 1A), suggesting that treatment effects on measured ecosystem variables may not be due to changes in species functional density but instead may reflect shifts in consumer behavior [23]. At this scale, manipulation treatment effects were only observed when evaluating above-ground biomass (Fig 1E).

Above-ground biomass was marginally higher in the Predator Exclusion treatment when compared to the control across all sites, but the Predator Addition treatment did not differ compared to either experimental treatment (Fig 1D). This is in opposition to original predictions in which a loss of predators would release herbivorous prey from top-down consumer control, leading to the overconsumption of above-ground vegetation. Three likely explanation for the observed results are that (1) an increase in burrowing behavior and crab excrement in cages without predators improved soil conditions enough for plant growth to counter losses caused by herbivory [48]; (2) that the rate of plant biomass production increased over the course of the experiment to compensate for losses due to unchecked herbivory [36]; and (3) the Predator Exclusion cages had larger stocks of initial above-ground biomass and production across the growing season maintained this difference between cages. Explanations 1–2 suggest that the recent studies indicating consumer control of primary production in salt marshes may not hold across all environmental contexts.

#### Farm River State Park

At Farm River State Park, burrow density did not differ in the Predator Exclusion treatment compared to the control but the Predator Addition treatment had marginally higher density relative to the control (Fig 2). The manipulation treatments did not differ from one another in burrow density or at any of the remaining response variables. An increase in burrow density within the Predator Addition treatment directly opposes initial predictions in which the presence of predators would reduce the functional density of burrowing crabs, thus reducing the number of burrows present. Prey species often shift their behavior in the presence of a predatory threat [23] and here, a shift towards a higher production of burrows to evade predation may explain these results.

SOM was significantly lower in both the Predator Exclusion and Predator Addition treatments relative to the control (Fig 2). The similar quantitative response for SOM for both manipulation types suggests that treatment effects did not drive these differences but that they may instead have arisen from cages limiting access to other non-target consumers. As a detritivore, fiddler crabs consume detritus and living microbial organisms, directly reducing soil organic matter content [49]. Further, burrowing activity increases soil drainage (i.e. liquid and gas exchange between the soil and environment), increasing soil oxidation, and thus improving conditions to favor the decomposition of organic debris [50, 51–54]. Both mechanisms may lead to a concomitant reduction in SOM. Additionally, cages may limit access to consumers that would positively influence SOM availability either via changes in mineralization rate or through direct inputs such as excrement. In each of these cases, the presence of experimental cages that fully restrict external consumer access would lead to both treatment plots containing less SOM relative to the control.

Finally, although there was no difference in soil inorganic nitrogen between the Predator Exclusion and control treatments, the Predator Addition treatment had marginally less soil nitrogen compared to the control and significantly less nitrogen compared to the Predator Exclusion treatment (Fig 2). This is consistent with the prediction that the presence of predators would lead to lower prey abundance and burrow production, causing an attendant reduction in soil inorganic nitrogen, but is inconsistent with the aforementioned burrow density and SOM results at this site. Therefore, it may instead be that, in the presence of predators, soil nitrogen is higher due to bioturbation as previously described, but the rate at which the enclosed vegetation takes up these nutrients is also higher [55]. Under these conditions, the amount of soil nitrogen available at the end of the experiment would then be less in the Predator Addition treatment relative to both control and Predator Exclusion treatments.

Overall results at this site indicate that ecosystem functions are variably influenced by top-down consumer control, but that this control is inconsistent across treatments and response variables. This suggests that there may be some context-dependency in how consumers impact large scale ecosystem processes.

#### Fence Creek

At Fence Creek, there was no treatment impact on burrow density, which again indicates that treatment effects on other measured variables may reflect behavioral shifts. Here, treatment effects were only observed for soil inorganic nitrogen content and above-ground biomass (Fig 2). Soil nitrogen was marginally higher in the Predator Exclusion treatment relative to the control, and significantly higher in the Predator Addition treatment compared to the control (Fig 2). Similar to Farm River State Park, this may reflect changes due to restricted access due to the cages and not experimental treatment effects, per se. If predators that consume fiddler and purple marsh crabs have limited access [56], an increase in burrowing behavior in both manipulation cages could lead to a greater amount of soil nitrogen via the ecosystem engineering and bioturbation mechanisms previously described. Above-ground biomass was marginally higher in the Predator Exclusion treatment relative to the control but did not differ in the Predator Addition treatment compared to the control (Fig 2). This may be explained by the same three mechanisms posited in the discussion of above-ground biomass across all sites combined [36, 48]. Overall, ecosystem functions at this site appear to be weakly correlated with consumer control via the crab food-chain evaluated here and is likely much more strongly influenced either by other non-target species or abiotic and bottom-up effects.

#### Hammonasset Beach State Park

At Hammonasset Beach State Park, burrow density was marginally lower in the Predator Addition treatment compared to both the control and Predator Exclusion treatments (Fig 2). These results follow from initial expectations that the presence of predators would reduce the functional density and burrowing behavior of the fiddler and purple marsh crabs. Here, the Predator Exclusion treatment had marginally more SOM compared to the control while the Predator Addition treatment had significantly more SOM relative to the control (Fig 2). More SOM suggests either reduced consumption of particulate organic matter or a decrease in decomposition, both of which can be due to a decline in fiddler and purple marsh crab abundance or shifts in behavior [49–54]. Casual observations at this site showed evidence of larger non-crustacean predator populations (e.g. egrets, night herons, etc), potentially altering prey abundance and behavior in addition to changes induced by experimental treatments [57]. Thus, consumption due to non-target predators that were not restricted to access to prey by cages may have influenced the increase in SOM observed in the Predator Exclusion cages while the additional stress of predators present in the Predator Addition cages may explain the larger SOM response.

Soil inorganic nitrogen was significantly higher in the Predator Exclusion treatment when compared to both the control and the Predator Addition treatment (Fig 2). This follows from original predictions but is not consistent with burrow density results at this site. This again may be due to the possibility of increased burrow production that is not captured in the data due to burrow collapse caused by the tidal regime. Additionally, an increase in soil nitrogen may be attributed to direct inputs in the form of crab excrements and the decomposition of SOM, which may have been predictably higher in cages where prey abundance was greater due to refuge from predators [28, 29, 58].

Lastly, the Predator Exclusion treatment had significantly more above-ground biomass compared to controls at Hammonasset but no other differences were observed among treatments (Fig 2). This may be explained by the same three mechanisms posited in the discussion of above-ground biomass across all sites combined [36, 48]. Overall results at this site indicate that consumers do have an effect on large scale ecosystem processes, but that their influence may be largely context-dependent.

#### Implications

Consumer interactions play a significant role in ecosystem functioning of ecosystems ranging from the rocky intertidal zone to arctic tundra systems [10–11, 21, 59–62]. These interactions regulate not only species functional density and behaviors, but also primary production, nutrient availability, and other dynamic ecosystem processes. Ecosystem functioning historically been thought to be driven primarily from bottom-up processes mediated through plant-soil interactions [16–19], but recent evidence suggests that consumers may play a much larger role in regulating functions and services under certain anthropogenic conditions [23–25]. With the caveat that these observations occurred over the course of one growing season, the results of this study build on growing evidence indicating that consumers may play an important role in regulating functions, but this control may be highly context-dependent.

In particular, neither the presence nor the absence of predators consistently impacted important variables such as above-ground biomass and soil quality across all sites; however, these measures were variably impacted when treatments were compared within each experimental site (Table 5). This suggests that, within these landscapes, both biotic and abiotic factors may impact the level of ecosystem functioning. Though less indicative of consistent control by the consumers evaluated here, changes observed in soil organic matter content and soil nitrogen availability expand on the current literature and indicate that consumer impacts are not confined to changes in biomass [20], but may also trickle down to impact additional larger-scale ecosystem processes.

As a critically important ecosystem throughout the world, wetlands provide services and benefits that other ecosystems do not. Thus, understanding the processes that regulate functions and processes within these systems is crucial. This study adds to growing literature indicating a need to evaluate wetland systems holistically, focusing not only the biological and ecological drivers of change, but also the contexts in which those changes are occurring. By doing so, we improve our ability to understand and safeguard the functions of these valuable and threatened ecosystems.

## Supporting information

S1 File All of the data collected from enclosure experiment. Burrow density units are number of burrows per square meter, soil inorganic nitrogen units are milligram nitrogen per gram of soil, rate of change in soil nitrogen (“Mineral”) units are milligram nitrogen per gram of soil per day, soil organic matter (“OM”) was measured as the proportion organic matter content per g of soil, and above-ground biomass units are gram per square meter.
(XLSX)

S1 Appendix R code used for all statistics for this project. This includes linear mixed models and GLMM to assess the effect of treatment on each response variable.
(R) Click here for additional data file.

S2 Appendix Results for ANOVA tests conducted to evaluate differences between the two non-manipulation control plots at each response variable with the exception of the rate of change in nitrogen. These data were not available for the non-manipulation cage control and were therefore not included in any analyses.
(DOCX)

S1 Table Initial survey of consumer and predator abundance by site. (DOCX)
