# Supplementary material for: Context-dependent consumer control in New England tidal wetlands
Source: PLoS One. 2018 May 17;13(5):e0197170. doi: 10.1371/journal.pone.0197170 (PMC5957357; doi:10.1371/journal.pone.0197170)
Supplement: S2 Appendix — These data were not available for the non-manipulation cage control and were therefore not included in any analyses. (DOCX) [file pone.0197170.s003.docx]

**ANOVA for Burrow Density**

Simultaneous Tests for General Linear Hypotheses

Multiple Comparisons of Means: Tukey Contrasts

Fit: aov (formula = Burrow_F ~ Trtmnt.f, data = AllSites2015)

Linear Hypotheses:

|  | Estimate | Std. Error | t value | Pr (>\|t\|) |
| --- | --- | --- | --- | --- |
| Control – Cage Control == 0 | 5.267 | 5.488 | 0.96 | 0.345 |

**ANOVA for Soil Organic Matter**

Simultaneous Tests for General Linear Hypotheses

Multiple Comparisons of Means: Tukey Contrasts

Fit: aov(formula = OM_F ~ Trtmnt.f, data = AllSites2015)

Linear Hypotheses:

|  | Estimate | Std. Error | t value | Pr (>\|t\|) |
| --- | --- | --- | --- | --- |
| Control – Cage Control == 0 | 0.02292 | 0.02857 | 0.802 | 0.428 |

**ANOVA for Soil Inorganic Nitrogen**

Simultaneous Tests for General Linear Hypotheses

Multiple Comparisons of Means: Tukey Contrasts

Fit: aov(formula = Nitrogen_F ~ Trtmnt.f, data = AllSites2015)

Linear Hypotheses:

|  | Estimate | Std. Error | t value | Pr (>\|t\|) |
| --- | --- | --- | --- | --- |
| Control – Cage Control == 0 | 0.0005916 | 0.0016967 | -0.349 | 0.73 |

**ANOVA for Biomass**

Simultaneous Tests for General Linear Hypotheses

Multiple Comparisons of Means: Tukey Contrasts

Fit: aov(formula = Biomass_F ~ Trtmnt.f, data = AllSites2015)

Linear Hypotheses:

|  | Estimate | Std. Error | t value | Pr (>\|t\|) |
| --- | --- | --- | --- | --- |
| Control – Cage Control == 0 | 60.19 | 65.11 | 0.924 | 0.363 |

*Note: Data for rate of change in nitrogen mineralization not available for non-manipulation cage control.
