# Supplementary material for: Context-dependent consumer control in New England tidal wetlands
Source: PLoS One. 2018 May 17;13(5):e0197170. doi: 10.1371/journal.pone.0197170 (PMC5957357; doi:10.1371/journal.pone.0197170)
Supplement: S1 Table — (DOCX) [file pone.0197170.s004.docx]

**Table S1. Initial survey of consumer and predator abundance by site.**

| Site | Fiddler Crab | Purple Marsh Crab | European Green Crab |
| --- | --- | --- | --- |
| Farm River State Park | 8.8 | 3.6 | 0.1 |
| Fence Creek marsh | 9.4 | 2.2 | 0.1 |
| Hammonasset Beach State Park | 11.2 | 3.8 | 0.2 |

Values represent average number of individuals per pitfall trap for fiddler and purple marsh crabs and average number of individuals per Quonset crab pot for the European green crab. Pitfall traps were constructed using 2.5-quart plastic buckets and 7.5 cm diameter open-top plastic cylinders (empty tennis ball cans) with drainage holes drilled in the bottom and sunk until the top edge was flush with the marsh substrate. Eight traps per site were placed >2m apart and left unbaited for 24 hours in May 2015 and checked the following day at low tide. Predator crabs were targeted using one baited and one unbaited Quonset crab pots placed adjacent to experimental treatment plots along the salt marsh creek edge where the tide was guaranteed to submerge the traps during the tidal regime. Pots were deployed overnight in each site on five separate occasions in May 2015.
